# Copper(II) Chelates of Schiff Bases Enriched with Aliphatic Fragments: Synthesis, Crystal Structure, In Silico Studies of ADMET Properties and a Potency against a Series of SARS-CoV-2 Proteins

**DOI:** 10.3390/ph16020286

**Published:** 2023-02-14

**Authors:** Elizaveta V. Panova, Julia K. Voronina, Damir A. Safin

**Affiliations:** 1 Institute of Chemistry, University of Tyumen, Volodarskogo Str. 6, 625003 Tyumen, Russia; 2 N.S. Kurnakov Institute of General and Inorganic Chemistry of the Russian Academy of Sciences, Leninsky Prospekt 31, GSP-1, 119991 Moscow, Russia; 3 Scientific and Educational and Innovation Center for Chemical and Pharmaceutical Technologies, Ural Federal University named after the First President of Russia B.N. Yeltsin, 620002 Ekaterinburg, Russia

**Keywords:** Schiff base, copper, synthesis, crystal structure, X-ray, ADMET, molecular docking, molecular dynamics, SARS-CoV-2, COVID-19

## Abstract

We report two complexes [Cu(L^I^)_2_] (**1**) and [Cu(L^II^)_2_] (**2**) (HL^I^ = *N*-cyclohexyl-3-methoxysalicylideneimine, HL^II^ = *N*-cyclohexyl-3-ethoxysalicylideneimine). The ligands in both complexes are *trans*-1,5-N,O-coordinated, yielding a square planar CuN_2_O_2_ coordination core. The molecule of **1** is planar with two cyclohexyl groups oriented to the opposite sites of the planar part of a molecule, while the molecule of **2** is significantly bent with two cyclohexyl groups oriented to the same convex site of a molecule. It was established that both complexes in MeOH absorb in the UV region due to intraligand transitions and LMCT. Furthermore, the UV-vis spectra of both complexes revealed two low intense shoulders in the visible region at about 460 and 520 nm, which were attributed to d–d transitions. Both complexes were predicted to belong to a fourth class of toxicity with the negative BBB property and positive gastrointestinal absorption property. According to the molecular docking analysis results, both complexes are active against all the applied SARS-CoV-2 proteins with the best binding affinity with Nsp 14 (N7-MTase), PLpro and Mpro. The obtained docking scores of complexes are either comparable to or even higher than those of the initial ligands. Complex **1** was found to be more efficient upon interaction with the applied proteins in comparison to complex **2**. Ligand efficiency scores for the initial ligands, **1** and **2** were also revealed.

## 1. Introduction

Copper is of great importance for living organisms since it plays a pivotal role in some biological processes. Particularly, a series of proteins comprise copper ions as prosthetic groups and are thus known as copper proteins [1,2], of which the metal-containing centers, in turn, are classified into several types. Of these types, Type II copper centers, abbreviated as T2Cu, contain a square planar coordination core formed either by the nitrogen- or mixed nitrogen/oxygen donor ligands [1]. T2Cu centers in the copper proteins are usually involved in redox processes [2].

Problems of health have plagued mankind throughout history. The most crucial problems concerning public health are, obviously, caused by diseases turned to pandemic, leading to hard-to-recover human and economic losses. Furthermore, previously unknown diseases further exacerbate the situation since neither efficient drugs nor therapies are known. Thus, the fabrication of novel compounds efficient against diseases is of great importance to overcome this fierce confrontation. World-wide attention has been focused during the last three years on one of the most fatal diseases in the modern history of mankind, namely severe acute respiratory syndrome-related coronavirus 2 (SARS-CoV-2), which is a causative of coronavirus disease 2019 (COVID-19). As a result, a pandemic was announced in March 2020 by the World Health Organization (WHO). To date, as of the middle of February 2023, about 755 million infections were confirmed with more than 6.8 million deaths [3]. The situation with COVID-19 remains complicated due to newly emerging strains. Thus, drugs against COVID-19 are of value.

Recently, researchers have directed their attention to copper to fight against COVID-19 [4,5,6]. Furthermore, in 1990, Lai et al., reported on substituted salicylaldehyde Schiff bases as new antiviral agents against coronavirus [7]. Lately, metal complexes of Schiff bases, including copper-based complexes, have also been in the limelight of studies to treat COVID-19 [8,9,10]. Thus, fabrication of copper-derived complexes with Schiff bases seems to be one of the strategies to produce active agents against COVID-19.

Schiff bases obtained from salicylaldehyde and its derivatives are, likely, the most abundant. These compounds, in the vast majority of cases, form N,O-chelated complexes with metal cations [11,12,13,14,15,16,17,18]. Thus, copper(II) complexes with such a type of Schiff bases also mimic prosthetic groups in copper proteins with the T2Cu centers due to the formation of the CuN_2_O_2_ coordination core. Notably, the CuN_2_O_2_ core was established for the oxidized active-site copper center of recombinant bifunctional peptidylglycine α-amidating enzyme [19]. This enzyme is responsible for the C-terminal peptide amidation, which is essential for the bioactivity of numerous peptide hormones involved in the regulation and control of cellular function.

We have also been interested in the chemistry of salicylaldehyde Schiff bases [20,21,22,23]. With all this in mind, as well as in continuation of our in silico studies of bioactive compounds [23,24,25], we have directed our attention to copper(II) complexes [Cu(L^I^)_2_] (**1**) and [Cu(L^II^)_2_] (**2**) (HL^I^ = *N*-cyclohexyl-3-methoxysalicylideneimine, HL^II^ = *N*-cyclohexyl-3-ethoxysalicylideneimine). It should be noted that a comprehensive search in the Cambridge Structural Database (CSD) [26] revealed only three hits of HL^I^-derived complexes with copper(II) [27], cobalt(II) [28] and nickel(II) [29] of the [M(L^I^)_2_] composition. Even more surprisingly, no crystal structures of metal complexes with HL^II^ have been reported so far. Although the crystal structure of **1** has already been known, as it was reported about 45 years ago [27]; herein, we have also revisited the crystal structure of **1** to solve it according to modern requirements and for a better comparison with the structure of **2**.

Bioavailability, druggability as well as absorption, distribution, metabolism, excretion and toxicity (ADMET) properties of both complexes were evaluated using a set of online tools. Using an in silico molecular docking method, we have explored the binding modes and interactions of **1** and **2** with binding sites of a series of the SARS-CoV-2 proteins. Ligand efficiency scores for the initial ligands, and complexes **1** and **2** inside the binding sites of the applied proteins were also revealed.

## 2. Results and Discussion

**Figure 1:**
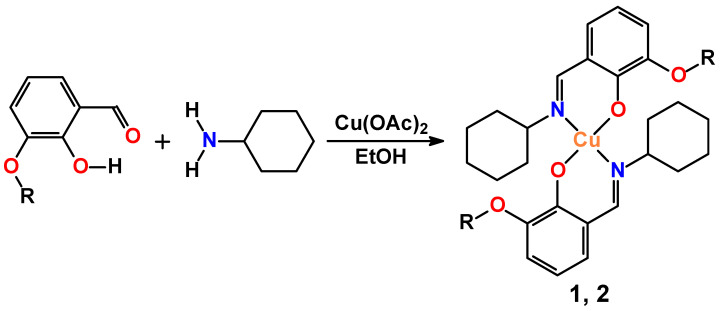
Synthesis of complexes (R = Me, **1**; Et, **2**).

A one pot in situ reaction of a solution of Cu(OAc)_2_ in ethanol with a solution of cyclohexylamine and 3-methoxy- or 3-ethoxysalicylaldehyde in the same solvent has facilitated the production of mononuclear discrete complexes [Cu(L^I^)_2_] (**1**) and [Cu(L^II^)_2_] (**2**) (HL^I^ = *N*-cyclohexyl-3-methoxysalicylideneimine, HL^II^ = *N*-cyclohexyl-3-ethoxysalicylideneimine), respectively (Figure 1). The isolated compounds were characterized by the means of the IR and UV-vis spectroscopy data. Their composition and structure were established by microanalysis, and single crystal and powder X-ray diffraction.

**Figure 2:**
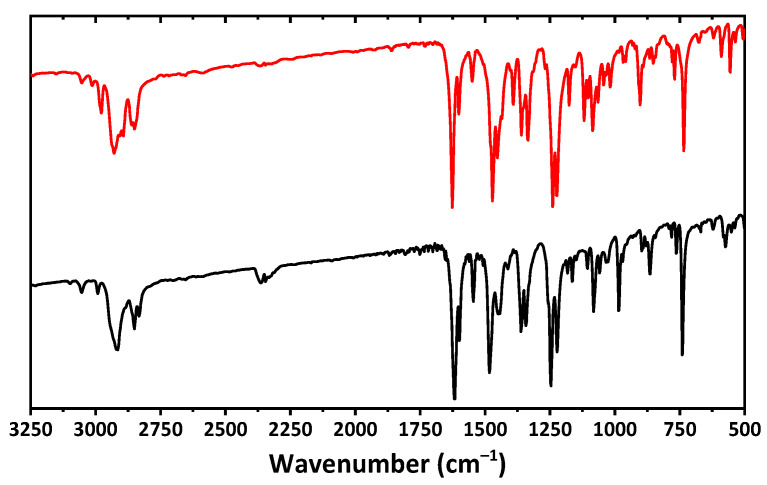
The IR spectra of **1** (black) and **2** (red) recorded in a KBr pellet.

The IR spectra of both complexes are very similar and contain a set of bands at about 2750–3100 cm^−1^ (Figure 2), corresponding to CH stretching vibrations of the aromatic and aliphatic fragments. An intense band at about 1620 cm^−1^ and a band at about 1600 cm^−1^ correspond to C=N and C=C bending. Bands at about 1360 and 1470–1480 cm^−1^ were attributed to CH stretching vibrations of the aliphatic groups. Vibrations of the C–O–C functionalities are shown as bands at about 1220–1250 cm^−1^.

**Figure 3:**
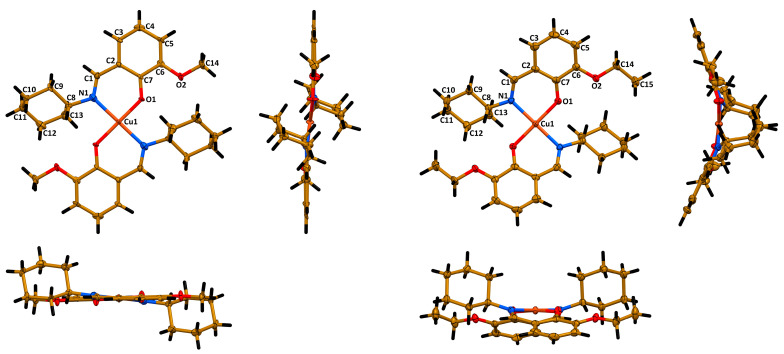
Different views of the molecular structures of **1** (left) and **2** (right). Ellipsoids are drawn with 50% probability. Color code: H = black, C = gold, N = blue, O = red, Cu = orange.

According to single crystal X-ray diffraction, **1** crystallizes in monoclinic space group *P*2_1_/*c* and the structure is the same as reported before [27]. Complex **2** crystallizes in orthorhombic space group *Pbcn*. The asymmetric unit cell of both complexes contains a half of a molecule [Cu(Lig)_2_] with the copper(II) cation lying on the inversion center. The ligands in both molecules are *trans*-coordinated through the imine nitrogen atom and phenolic oxygen atom, yielding a square planar CuN_2_O_2_ coordination core with two Cu1~N1~C1~C2~C7~O1 six-membered chelate rings (Figure 3). All five atoms of the coordination core are perfectly lying on the same least square plane in the structure of **1**, while in the structure of **2** these atoms are slightly deviated from the least square plane (Cu1~0.02 Å, N1~0.04 Å, O1~0.05 Å). The Cu1–N1/O1, C1–N1 and C7–O1 bond lengths and bond angles around the metal cation are similar in both structures (Table 1). However, a minor deviation from linearity was observed for the O1–Cu1–O1′ bond angle in the structure of **2** (Table 1). Notably, the cyclohexyl fragments in both structures adopt a chair conformation (Figure 3).

Interestingly, the most crucial difference between the molecular structures of the described complexes was observed for the overall geometry of the molecules. Particularly, the molecule of **1** is essentially planar with two cyclohexyl groups oriented to the opposite sites of the planar part of a molecule (Figure 3). However, the molecule of **2** is significantly bent with two cyclohexyl groups oriented to the same convex site of a molecule (Figure 3). This is also clearly reflected from the corresponding dihedral angles (Table 1). From one side, such a dramatic difference in the molecular structures of complexes can be explained by a repulsion of the bulky cyclohexyl fragment and the methyl group of the ethoxy fragment in the structure. However, from the other side, closer inspection and comparison of the molecular structures of **1** and **2** has allowed us to reveal that the cyclohexyl fragments tend to form C–H∙∙∙H–C homopolar dihydrogen bonding with the imine hydrogen atoms, methyl hydrogen atoms and oxygen atoms of the methoxy fragment in **1** and ethoxy fragment in **2**. Recently, we have reported on the influence of C–H∙∙∙H–C homopolar dihydrogen bonding on the overall stabilization of the molecular structure of coordination compounds and even on the crucial influence of this interaction on coordination geometry [30]. In-depth studies of intramolecular interactions in the molecular structures of complexes **1** and **2** will be performed using computational approaches and the obtained results will be published elsewhere.

**Figure 4:**
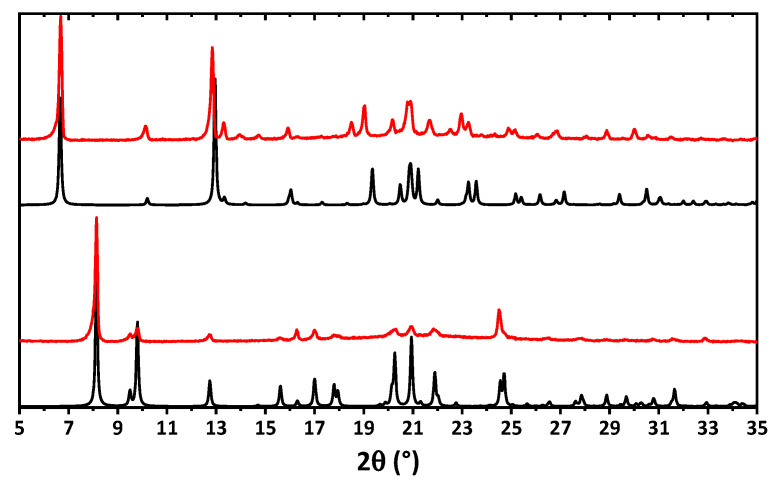
Calculated (black) and experimental (red) powder X-ray diffraction patterns of **1** (bottom) and **2** (top).

The bulk samples of **1** and **2** were examined by means of powder X-ray diffraction analysis (Figure 4). The experimental X-ray powder pattern is in full agreement with the calculated powder pattern obtained from single crystal X-ray diffraction, showing that the bulk material is free from phase impurities.

**Figure 5:**
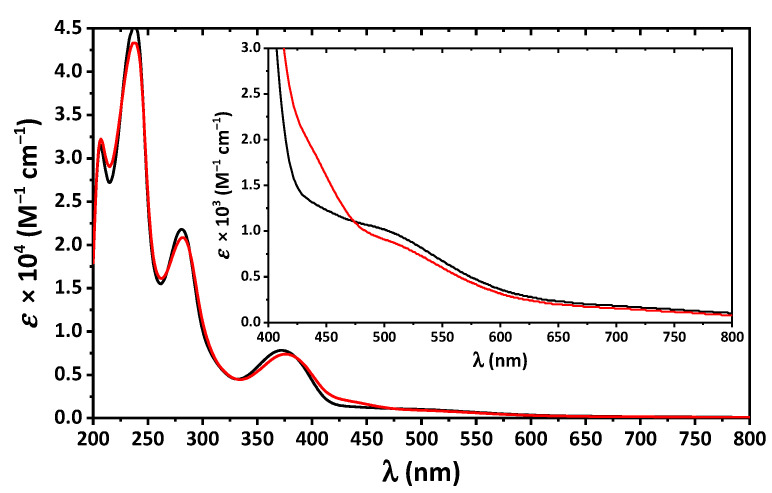
The UV-vis spectra of **1** (black) and **2** (red) in MeOH.

The absorption spectra of **1** and **2** in MeOH are very similar and contain bands up to about 650 nm with four clearly defined maxima at about 205, 240, 280 and 375 nm (Figure 5). The former high-energy bands correspond to intraligand π → π* and n → π* transitions arising from the benzene and imine fragments, while the latter band was assigned to ligand-to-metal charge transfer (LMCT). Furthermore, a closer inspection of the UV-vis spectra of both complexes revealed two low intense shoulders in the visible region at about 460 and 520 nm, which were attributed to d–d transitions (Figure 5).

**Figure 6:**
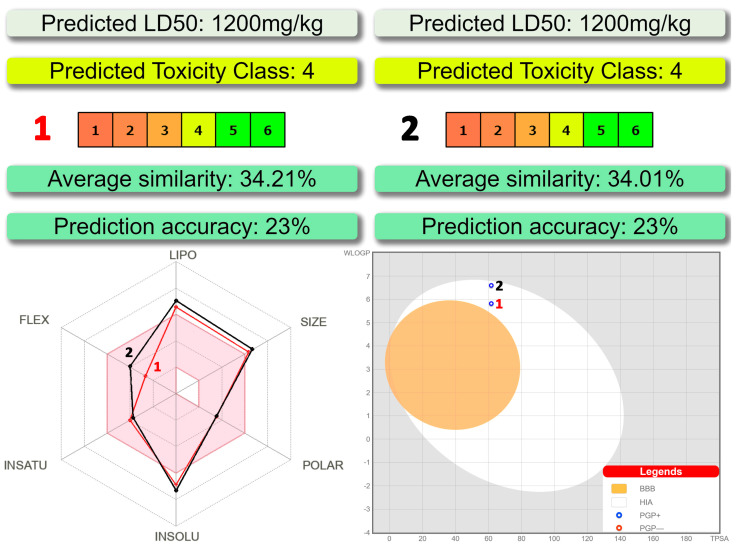
(top) Toxicity results of **1** and **2** calculated by ProTox-II. (bottom left) Bioavailability radar for **1** and **2** within the domain borders of ADME properties, calculated by SwissADME. The colored zone of the radar is the suitable physicochemical space for oral bioavailability. (bottom right) BOILED-Egg model of **1** and **2** calculated by SwissADME.

According to ProTox-II, a virtual lab for the prediction of toxicities of small molecules [31,32], both complexes belong to a fourth class of toxicity with the predicted LD_50_ of about 1200 mg/kg (Figure 6). As evidenced from the SwissADME [33] bioavailability radar, the discussed compounds are preferred in the three parameters, namely polarity, insaturation and flexibility, and less preferred in lipophilicity, size and insolubility (Figure 6).

The BOILED-Egg method was found to be efficient to predict the human blood-brain barrier (BBB) penetration and gastrointestinal absorption [34]. This approach is based on lipophilicity (WLOGP) and polarity (topological polar surface area, TPSA) (Figure 6). Points located in the yellow region (BOILED-Egg’s yolk) are molecules predicted to passively permeate through the BBB, while points located in the white region (BOILED Egg’s white) are molecules predicted to be passively absorbed by the gastrointestinal tract. Blue (PGP+) and red (PGP−) dots are for molecules predicted to be effluated and not to be effluated from the central nervous system by the P-glycoprotein, respectively. As evidenced from the blue dots’ positions for both complexes, the BBB penetration property is negative and gastrointestinal absorption property is positive with the positive PGP effect on the molecule (Figure 6).

We have further applied a molecular docking approach for both complexes with a series of the SARS-CoV-2 proteins. Furthermore, initial ligands were also redocked for a proper comparison of the obtained results. The target structures were primarily selected in accordance with the structural features of the virus [35,36] as well as based on biological mechanisms and functions that can be utilized to reduce, prevent or treat the virus [37] (Table 2).

**Figure 7:**
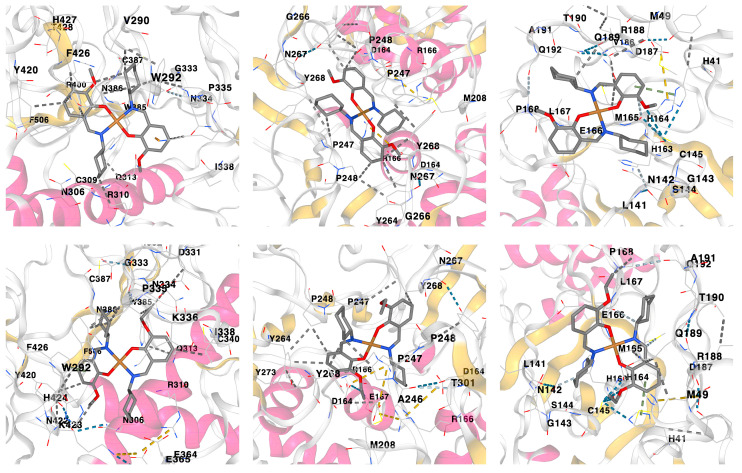
Three-dimensional views on the interaction of complexes **1** (top) and **2** (bottom) with (from left to right) Nonstructural protein 14 (N7-MTase), Papain-like protease (PLpro) and Main protease (Mpro).

According to the docking analysis results, both complexes were found to be active against all the applied SARS-CoV-2 proteins with the best binding affinity with Nonstructural protein 14 (N7-MTase), Papain-like protease (PLpro) and Main protease (Mpro) (Figure 7, Table 3). Furthermore, the obtained docking scores of complexes are either comparable to or even higher of those of the initial ligands (Table 3). Moreover, complex **1** was found to be more efficient upon interaction with the applied proteins in comparison to complex **2** (Table 3). Interactions responsible for binding of **1** and **2** with Nonstructural protein 14 (N7-MTase), Papain-like protease (PLpro) and Main protease (Mpro) are shown in Figure 7 and collected in Table 2. According to the obtained results, hydrophobic interactions of the alkyl and π∙∙∙alkyl types are main contributors for binding the ligands to proteins (Table 3).

We have also established additional ligand efficiency scores to shed more light on the bioactivity of **1** and **2** towards the applied SARS-CoV-2 proteins. As such, for all complexes of **1** and **2** with the studied proteins, we have calculated inhibition constant (*K_i_*), miLogP, ligand efficiency (LE), ligand efficiency_scale (LE_Scale), fit quality (FQ) and ligand-efficiency-dependent lipophilicity (LELP) [38,39,40,41,42,43] (Table 2). Furthermore, for comparison we have also calculated the same ligand efficiency scores for complexes of the studied proteins with initial ligands (Table 2). Notably, the *K_i_* value must be as low as possible for a more efficient inhibition and should fall in the μM range for a compound to be considered as a Hit, and >10 nM for a drug [42]. Furthermore, for a compound to be considered as a Hit the LE, FQ and LELP parameters are recommended as ≥0.3, ≥0.8 and from −10 to 10, respectively [42].

Of all the complexes of the applied proteins with **1** and **2**, the ligand efficiency scores for complexes with Nonstructural protein 14 (N7-MTase) are close to be within the recommended ranges for a Hit and even close to values for a drug, although the LELP values are clearly out of the recommended range (Table 2). These results are preferable for complex of Nonstructural protein 14 (N7-MTase) with **1** in comparison to **2** and even to the initial ligand, although the *K_i_* value for a latter ligand is about two times lower but with a less preferable LELP value (Table 2). For complexes of **1** with Papain-like protease (PLpro) and Main protease (Mpro), the ligand efficiency scores are also within the recommended ranges and even superior to those of the initial ligand except for the LELP values (Table 2).

## 3. Materials and Methods

### 3.1. Physical Measurements

The IR spectra in KBr pellets were recorded with a FT-IR FSM 1201 spectrometer in the range 400–4000 cm^−1^. UV–vis spectra from the 10^−4^ M freshly prepared solutions in freshly distilled MeOH were recorded on an Agilent 8453 instrument. Powder X-ray diffraction was carried out using a Rigaku Ultima IV X-ray powder diffractometer. The parallel beam mode was used to collect the data (λ = 1.54184 Å). Elemental analyses were performed with a Thermo Scientific FLASH 2000 CHNS analyzer (Waltham, MA USA).

### 3.2. Synthesis

A hot solution of Cu(OAc)_2_ (1 mmol, 0.182 g) in ethanol (10 mL) was added dropwise to a hot solution of cyclohexylamine (2 mmol, 0.198 g) and 3-methoxysalicylaldehyde or 3-ethoxysalicylaldehyde (2 mmol, 0.304 and 0.332 g) in the same solvent (20 mL) under vigorous stirring. The resulting mixture was left undisturbed under ambient conditions for slow evaporation of the solvent to give green prism-like crystals suitable for single crystal X-ray diffraction.

**Complex 1.** Yield: 0.481 g (91%). Anal. Calc. for C_28_H_36_CuN_2_O_4_ (528.15): C 63.68, H 6.87 and N 5.30; found: C 63.77, H 6.92 and N 5.24%.

**Complex 2.** Yield: 0.462 g (83%). Anal. Calc. for C_30_H_40_CuN_2_O_4_ (556.20): C 64.78, H 7.25 and N 5.04; found: C 64.70, H 7.33 and N 4.99%.

### 3.3. Single Crystal X-ray Diffraction

The X-ray diffraction data for **1** and **2** were collected on Bruker SMART Apex-II and Bruker D8 Venture diffractometers, respectively, equipped with a CCD detector (Mo-Kα, λ = 0.71073 Å, graphite monochromator). Semi-empirical absorption correction was applied by the SADABS program [44]. The structures were solved by direct methods and refined by the full-matrix least squares in the anisotropic approximation for non-hydrogen atoms. The structure of **1** was refined as a two-component twin. The calculations were carried out by the SHELX-2014 program package [45] using Olex2 1.2 [46]. CCDC 2235309 and 2235310 contain the crystallographic data for **1** and **2**, respectively. These data can be obtained free of charge via https://www.ccdc.cam.ac.uk/structures (accessed on 7 February 2023) or from the Cambridge Crystallographic Data Centre, 12 Union Road, Cambridge CB2 1EZ, UK; fax: (+44)-1223-336-033; or e-mail: deposit@ccdc.cam.ac.uk.

**Crystal data of 1.** C_28_H_36_CuN_2_O_4_, *Mr* = 528.13 g mol^−1^, *T* = 150(2) K, monoclinic, space group *P*2_1_/*c*, *a* = 11.0369(16), *b* = 17.743(4), *c* = 6.3402(13) Å, *β* = 99.839(9)°, *V* = 1223.3(4) Å^3^, *Z* = 2, *ρ* = 1.434 g cm^−3^, *μ*(Mo-Kα) = 0.931 mm^−1^, reflections: 2397 collected, 2397 unique, *R*_int_ = 0.000, *R*_1_(all) = 0.1132, *wR*_2_(all) = 0.2136, *S* = 1.021.

**Crystal data of 2.** C_30_H_40_CuN_2_O_4_, *Mr* = 556.18 g mol^−1^, *T* = 100(2) K, orthorhombic, space group *Pbcn*, *a* = 26.5364(13), *b* = 9.1654(4), *c* = 11.0988(7) Å, *V* = 2699.4(2) Å^3^, *Z* = 4, *ρ* = 1.369 g cm^−3^, *μ*(Mo-Kα) = 0.847 mm^−1^, reflections: 26,069 collected, 3597 unique, *R*_int_ = 0.077, *R*_1_(all) = 0.0693, *wR*_2_(all) = 0.0963, *S* = 1.035.

### 3.4. Molecular Docking

Molecular docking simulations of complexes **1** and **2** with a series of the SARS-CoV-2 proteins were carried using the CB-Dock2 server [47,48], which reveals protein cavities to guide blind docking by the algorithm of AutoDock Vina [49]. The targeted protein structures were subtracted from the RCSB PDB database [50] and were pretreated before the docking, including water removing and inserting hydrogen atoms and missing residues and charges.

### 3.5. In Silico Drug-Likeness Analysis

Bioavailability, druggability as well as absorption, distribution, metabolism, excretion and toxicity properties were evaluated using the SwissADME [33], BOILED-Egg [34] and ProTox-II [31,32] tools.

## 4. Conclusions

We have synthesized complexes [Cu(L^I^)_2_] (**1**) and [Cu(L^II^)_2_] (**2**) (HL^I^ = *N*-cyclohexyl-3-methoxysalicylideneimine, HL^II^ = *N*-cyclohexyl-3-ethoxysalicylideneimine), which were confirmed by IR spectroscopy, single crystal and powder X-ray diffraction, and elemental analysis. The ligands in both complexes are *trans*-1,5-N,O-coordinated, yielding a square planar CuN_2_O_2_ coordination core. The molecule of **1** is essentially planar with two cyclohexyl groups oriented to the opposite sites of the planar part of a molecule, while the molecule of **2** is significantly bent with two cyclohexyl groups oriented to the same convex site of a molecule. Complexes in MeOH absorb in the UV region due to intraligand transitions and LMCT. Furthermore, the UV-vis spectra of **1** and **2** revealed two low intense shoulders in the visible region at about 460 and 520 nm due to d–d transitions.

Both complexes were predicted to belong to a fourth class of toxicity with the negative BBB property and positive gastrointestinal absorption property and the positive PGP effect on the molecule. Complexes were also found to be active against all the applied SARS-CoV-2 proteins with the best binding affinity with Nsp 14 (N7-MTase), PLpro and Mpro. The obtained docking scores of complexes are either comparable to or even higher of those of the initial ligands. Finally, complex **1** was found to be more efficient upon interaction with the applied proteins in comparison to complex **2**. Ligand efficiency scores for complexes of **1** and **2** with Nsp 14 (N7-MTase) are close to being within the recommended ranges for a Hit and even close to the values required for a drug.

## Figures and Tables

**Table 1:** Selected bond lengths (Å), and bond and dihedral angles (°) in the crystal structures of **1** and **2**.

	1	2		1	2
Bond length					
Cu1–N1	2.036(5)	2.0008(17)	C1–N1	1.292(7)	1.287(3)
Cu1–O1	1.875(4)	1.8970(13)	C1–O1	1.311(7)	1.304(2)
Bond angle					
N1–Cu1–O1	91.55(19)	91.19(6)	N1–Cu1–N1′	180.00	179.00(6)
N1–Cu1–O1′	88.45(19)	88.85(6)	O1–Cu1–O1′	180.00	175.27(6)
Dihedral angle					
N1–Cu1–O1–C1	8.3(5)	25.20(16)	O1–Cu1–N1′–C1′	−170.4(5)	−153.51(15)
O1–Cu1–N1–C1	−9.6(5)	−21.76(15)	C_6_H_3_∙∙∙C_6_H_3_	0.00	44.35
N1–Cu1–O1′–C′1	171.7(5)	155.80(16)			

**Table 2:** Ligand efficiency scores for the initial ligands, and complexes **1** and **2** inside the binding sites of the listed proteins.

Ligand Efficiency Score	Initial Ligand *	1	2
Main protease (Mpro) (PDB code 6LU7)			
Binding energy (BE, kcal/mol)	−7.4(1)	−8.6(0)	−7.5(1)
Inhibition constant (*K_i_* = *e*^(−BE/*RT*)^, μM) **	3.76	0.50	3.18
miLogP	2.32	5.37	6.13
Ligand efficiency (LE = −BE/(Heavy atoms), kcal/(mol HA)	0.151	0.246	0.203
LE_Scale (0.0715 + 7.5328/(HA) + 25.7079/(HA^2^) − 361.4722/(HA^3^))	0.233	0.299	0.287
Fit quality (FQ = LE/LE_Scale)	0.649	0.821	0.707
Ligand-efficiency-dependent lipophilicity (LELP = miLogP/LE)	15.362	21.855	30.241
Papain-like protease (PLpro) (PDB code 6WUU)			
Binding energy (BE, kcal/mol)	−8.6(1)	−8.7(0)	−7.9(0)
Inhibition constant (*K_i_* = *e*^(−BE/*RT*)^, μM) **	0.50	0.42	1.62
miLogP	−1.61	5.37	6.13
Ligand efficiency (LE = −BE/(Heavy atoms), kcal/(mol HA)	0.239	0.249	0.214
LE_Scale (0.0715 + 7.5328/(HA) + 25.7079/(HA^2^) − 361.4722/(HA^3^))	0.293	0.299	0.287
Fit quality (FQ = LE/LE_Scale)	0.816	0.831	0.745
Ligand-efficiency-dependent lipophilicity (LELP = miLogP/LE)	−6.740	21.603	28.710
Nonstructural protein 3 (Nsp3_range 207–379-AMP) (PDB code 6W6Y)			
Binding energy (BE, kcal/mol)	−7.2(0)	−7.4(1)	−7.5(1)
Inhibition constant (*K_i_* = *e*^(−BE/*RT*)^, μM) **	5.28	3.76	3.18
miLogP	−1.52	5.37	6.13
Ligand efficiency (LE = −BE/(Heavy atoms), kcal/(mol HA)	0.313	0.211	0.203
LE_Scale (0.0715 + 7.5328/(HA) + 25.7079/(HA^2^) − 361.4722/(HA^3^))	0.418	0.299	0.287
Fit quality (FQ = LE/LE_Scale)	0.749	0.706	0.707
Ligand-efficiency-dependent lipophilicity (LELP = miLogP/LE)	−4.856	25.399	30.241
Nonstructural protein 3 (Nsp3_range 207–379-MES) (PDB code 6W6Y)			
Binding energy (BE, kcal/mol)	−5.8(0)	−7.7(0)	−7.4(0)
Inhibition constant (*K_i_* = *e*^(−BE/*RT*)^, μM) **	56.05	2.27	3.76
miLogP	−4.08	5.37	6.13
Ligand efficiency (LE = −BE/(Heavy atoms), kcal/(mol HA)	0.483	0.220	0.200
LE_Scale (0.0715 + 7.5328/(HA) + 25.7079/(HA^2^) − 361.4722/(HA^3^))	0.669	0.299	0.287
Fit quality (FQ = LE/LE_Scale)	0.723	0.735	0.698
Ligand-efficiency-dependent lipophilicity (LELP = miLogP/LE)	−8.441	24.409	30.650
RdRp-RNA (PDB code 7BV2)			
Binding energy (BE, kcal/mol)	−6.6(0)	−7.2(0)	−6.6(0)
Inhibition constant (*K_i_* = *e*^(−BE/*RT*)^, μM) **	14.53	5.28	14.53
miLogP	−1.55	5.37	6.13
Ligand efficiency (LE = −BE/(Heavy atoms), kcal/(mol HA)	0.264	0.206	0.178
LE_Scale (0.0715 + 7.5328/(HA) + 25.7079/(HA^2^) − 361.4722/(HA^3^))	0.391	0.299	0.287
Fit quality (FQ = LE/LE_Scale)	0.676	0.687	0.622
Ligand-efficiency-dependent lipophilicity (LELP = miLogP/LE)	5.871	26.104	34.365
Nonstructural protein 14 (N7-MTase) (PDB code 5C8S)			
Binding energy (BE, kcal/mol)	−10.7(0)	−10.4(0)	−9.6(0)
Inhibition constant (*K_i_* = *e*^(−BE/*RT*)^, μM) **	0.01	0.02	0.09
miLogP	−4.67	5.37	6.13
Ligand efficiency (LE = −BE/(Heavy atoms), kcal/(mol HA)	0.214	0.297	0.259
LE_Scale (0.0715 + 7.5328/(HA) + 25.7079/(HA^2^) − 361.4722/(HA^3^))	0.230	0.299	0.287
Fit quality (FQ = LE/LE_Scale)	0.932	0.993	0.905
Ligand-efficiency-dependent lipophilicity (LELP = miLogP/LE)	−21.822	18.072	23.626
Nonstructural protein 15 (endoribonuclease) (PDB code 6WLC)			
Binding energy (BE, kcal/mol)	−7.5(1)	−7.8(0)	−7.6(0)
Inhibition constant (*K_i_* = *e*^(−BE/*RT*)^, μM) **	3.18	1.92	2.69
miLogP	−2.76	5.37	6.13
Ligand efficiency (LE = −BE/(Heavy atoms), kcal/(mol HA)	0.357	0.223	0.205
LE_Scale (0.0715 + 7.5328/(HA) + 25.7079/(HA^2^) − 361.4722/(HA^3^))	0.449	0.299	0.287
Fit quality (FQ = LE/LE_Scale)	0.795	0.745	0.716
Ligand-efficiency-dependent lipophilicity (LELP = miLogP/LE)	−7.728	24.096	29.843
Nonstructural protein 16 (GTA site) (PDB code 6WVN)			
Binding energy (BE, kcal/mol)	−8.7(1)	−7.7(0)	−6.9(0)
Inhibition constant (*K_i_* = *e*^(−BE/*RT*)^, μM) **	0.42	2.27	8.75
miLogP	−5.69	5.37	6.13
Ligand efficiency (LE = −BE/(Heavy atoms), kcal/(mol HA)	0.171	0.220	0.186
LE_Scale (0.0715 + 7.5328/(HA) + 25.7079/(HA^2^) − 361.4722/(HA^3^))	0.226	0.299	0.287
Fit quality (FQ = LE/LE_Scale)	0.754	0.735	0.650
Ligand-efficiency-dependent lipophilicity (LELP = miLogP/LE)	−33.355	24.409	32.871
Nonstructural protein 16 (MGP site) (PDB code 6WVN)			
Binding energy (BE, kcal/mol)	−6.7(0)	−6.3(0)	−6.3(1)
Inhibition constant (*K_i_* = *e*^(−BE/*RT*)^, μM) **	12.27	24.10	24.10
miLogP	−4.22	5.37	6.13
Ligand efficiency (LE = −BE/(Heavy atoms), kcal/(mol HA)	0.203	0.180	0.170
LE_Scale (0.0715 + 7.5328/(HA) + 25.7079/(HA^2^) − 361.4722/(HA^3^))	0.313	0.299	0.287
Fit quality (FQ = LE/LE_Scale)	0.648	0.601	0.594
Ligand-efficiency-dependent lipophilicity (LELP = miLogP/LE)	−20.785	29.833	36.002
Nonstructural protein 16 (SAM site) (PDB code 6WVN)			
Binding energy (BE, kcal/mol)	−7.3(1)	−7.2(1)	−7.3(1)
Inhibition constant (*K_i_* = *e*^(−BE/*RT*)^, μM) **	4.46	5.28	4.46
miLogP	−5.01	5.37	6.13
Ligand efficiency (LE = −BE/(Heavy atoms), kcal/(mol HA)	0.270	0.206	0.197
LE_Scale (0.0715 + 7.5328/(HA) + 25.7079/(HA^2^) − 361.4722/(HA^3^))	0.367	0.299	0.287
Fit quality (FQ = LE/LE_Scale)	0.736	0.687	0.688
Ligand-efficiency-dependent lipophilicity (LELP = miLogP/LE)	−18.530	26.104	31.070

* (from top to bottom) Initial ligand = *N*-[(5-methylisoxazol-3-yl)carbonyl]alanyl-*L*-valyl-*N*~1~-((1*R*,2*Z*)-4-(benzyloxy)-4-oxo-1-{[(3*R*)-2-oxopyrrolidin-3-yl]methyl}but-2-enyl)-*L*-leucinamide; methyl 4-[2-[[(2~{*S*})-2-[[(2~{*S*})-2-acetamido-4-(1,3-benzothiazol-2-yl)butanoyl]amino]-3-azanyl-propanoyl]amino]ethanoylamino]butanoate; adenosine monophosphate; 2-morpholin-4-ium-4-ylethanesulfonate; [(2~{*R*},3~{*S*},4~{*R*},5~{*R*})-5-(4-azanylpyrrolo [2,1-f][1,2,4]triazin-7-yl)-5-cyano-3,4-*bis*(oxidanyl)oxolan-2-yl]methyl dihydrogen phosphate; [(2R,3S,4R,5R)-5-(2-amino-6-oxo-1H-purin-9-yl)-3,4-dihydroxy-oxolan-2-yl]methyl [[[(2R,3S,4R,5R)-5-(6-aminopurin-9-yl)-3,4-dihydroxy-oxolan-2-yl]methoxy-hydroxy-phosphoryl]oxy-hydroxy-phosphoryl] hydrogen phosphate; uridine-5′-monophosphate; [(2R,3S,4R,5R)-5-(2-amino-7-methyl-6-oxo-1H-purin-7-ium-9-yl)-3,4-dihydroxy-oxolan-2-yl]methyl [[[(2R,3S,4R,5R)-5-(6-aminopurin-9-yl)-3,4-dihydroxy-oxolan-2-yl]methoxy-hydroxy-phosphoryl]oxy-hydroxy-phosphoryl] hydrogen phosphate; 7-methyl-guanosine-5′-triphosphate; *S*-adenosylmethionine. ** *R* = 1.9872 × 10^−3^ kcal/(mol K), *T* = 298.15 K.

**Table 3:** The best types of interactions and distances of complexes **1** and **2** with Nonstructural protein 14 (N7-MTase), Papain-like protease (PLpro) and Main protease (Mpro).

Interaction	Distance (Å)	Bonding	Bonding Type
Nonstructural protein 14 (N7-MTase)–**1**			
D:CYS309–A:1	4.55383	Hydrophobic	Alkyl
D:ARG310–A:1:C11	5.03726	Hydrophobic	Alkyl
D:TRP292–A:1:C13	5.40534	Hydrophobic	π∙∙∙Alkyl
D:TYR420–A:1:C4′	5.06410	Hydrophobic	π∙∙∙Alkyl
D:PHE426–A:1	3.96141	Hydrophobic	π∙∙∙Alkyl
Papain-like protease (PLpro)–**1**			
A:PRO248–A:1:C4	3.78832	Hydrophobic	Alkyl
C:PRO248–A:1:C5′	4.73701	Hydrophobic	Alkyl
C:TYR264–A:1:C5′	4.72694	Hydrophobic	π∙∙∙Alkyl
Main protease (Mpro)–**1**			
A:1:C4′–A:HIS41	3.74205	Hydrophobic	π∙∙∙Sigma
A:CYS145–A:1	4.99879	Hydrophobic	Alkyl
A:1:C2–A:MET165	4.58696	Hydrophobic	Alkyl
Nonstructural protein 14 (N7-MTase)–**2**			
A:2:C15–D:ASN334	3.58506	Hydrogen Bond	Carbon Hydrogen Bond
A:2:C15–D:TRP385:O	3.55155	Hydrogen Bond	Carbon Hydrogen Bond
D:PRO335–A:2:C12	5.22535	Hydrophobic	Alkyl
A:2:C15′–D:LYS423	4.53131	Hydrophobic	Alkyl
D:TYR420–A:2:C5	5.24129	Hydrophobic	π∙∙∙Alkyl
D:PHE426–A:2:C3	5.37133	Hydrophobic	π∙∙∙Alkyl
A:2:C15–D:ASN334	4.03087	Hydrophobic	π∙∙∙Alkyl
A:2:C15–D:TRP385:O	5.14456	Hydrophobic	π∙∙∙Alkyl
Papain-like protease (PLpro)–**2**			
A:PRO247–A:2:C13	5.16136	Hydrophobic	Alkyl
A:PRO247–A:2	5.14188	Hydrophobic	Alkyl
A:PRO248–A:2	4.46827	Hydrophobic	Alkyl
C:PRO248–A:2	4.76755	Hydrophobic	Alkyl
A:2:C14–A:MET208	4.24574	Hydrophobic	Alkyl
C:TYR264–A:2:C4	4.56104	Hydrophobic	π∙∙∙Alkyl
Main protease (Mpro)–**2**			
A:CYS145–A:2:O2	5.13188	Hydrophobic	Alkyl
A:MET165–A:2:C1′	5.06805	Hydrophobic	Alkyl
A:PRO168–A:2:C15	5.23131	Hydrophobic	Alkyl
A:2:C15′–A:PRO168	4.16653	Hydrophobic	Alkyl
A:2–A:MET49	5.08585	Hydrophobic	Alkyl
A:2–A:CYS145	4.80241	Hydrophobic	Alkyl
A:HIS41–A:2:C2	4.63051	Hydrophobic	π∙∙∙Alkyl
A:HIS41–A:2:C15	3.89620	Hydrophobic	π∙∙∙Alkyl

## Data Availability

Data is contained within the article.
